# A comparative proteomic study identified LRPPRC and MCM7 as putative actors in imatinib mesylate cross-resistance in Lucena cell line

**DOI:** 10.1186/1477-5956-10-23

**Published:** 2012-03-30

**Authors:** Stephany Corrêa, Luciana Pizzatti, Bárbara Du Rocher, André Mencalha, Daniela Pinto, Eliana Abdelhay

**Affiliations:** 1 Laboratório Célula-Tronco, Divisão de Laboratórios do CEMO, Instituto Nacional de Câncer, Rio de Janeiro, Brazil; 2 Instituto de Biofísica Carlos Chagas Filho, Universidade Federal do Rio de Janeiro, Rio de Janeiro, Brazil; 3 Instituto Nacional de Câncer, Praça da Cruz Vermelha, n° 23, 6° andar, ala C, CEMO, Laboratório Célula-Tronco, CEP: 20230130 Rio de Janeiro, RJ, Brazil

**Keywords:** Imatinib mesylate, Chronic myeloid leukemia, LRPPRC, MCM7, Proteome, Mass spectrometry

## Abstract

**Background:**

Although chronic myeloid leukemia (CML) treatment has improved since the introduction of imatinib mesylate (IM), cases of resistance have been reported. This resistance has been associated with the emergence of multidrug resistance (MDR) phenotype, as a BCR-ABL independent mechanism. The classic pathway studied in MDR promotion is ATP-binding cassette (ABC) family transporters expression, but other mechanisms that drive drug resistance are largely unknown. To better understand IM therapy relapse due to the rise of MDR, we compared the proteomic profiles of K562 and Lucena (K562/VCR) cells.

**Results:**

The use of 2-DE coupled with a MS approach resulted in the identification of 36 differentially expressed proteins. Differential mRNA levels of *leucine-rich PPR motif-containing (LRPPRC) protein, minichromosome maintenance complex component 7 (MCM7) *and *ATP-binding cassette sub-family B (MDR/TAP) member 1 (ABCB1) *were capable of defining samples from CML patients as responsive or resistant to therapy.

**Conclusions:**

Through the data presented in this work, we show the relevance of MDR to IM therapy. In addition, our proteomic approach identified candidate actors involved in resistance, which could lead to additional information on BCR-ABL-independent molecular mechanisms.

## Background

Chronic myeloid leukemia (CML) is a myeloproliferative disease, and the BCR-ABL constitutive tyrosine kinase (TK), an oncoprotein, serves as a marker of this condition. This oncoprotein is responsible for the pathogenesis of CML and is the primary molecular target for disease therapy [1]. Although CML treatment has improved with the development of imatinib mesylate (IM, Glivec^®^, Gleevec - Novartis), a TK inhibitor, some patients fail to respond to this therapy [2]. Resistance to treatment was first related to BCR-ABL-dependent mechanisms, such as mutations of the BCR-ABL kinase site. However, mutations are not seen in all patients who are resistant to IM treatment. This suggests that other resistance mechanisms occur in these patients. Among these mechanisms, known as BCR-ABL-independent mechanisms, is the multidrug resistance (MDR) phenotype [3-9]. MDR is known to be the major cause of failure in cancer treatment and has a multifactorial origin. It is related to the expression of ATP-binding cassette (ABC) family transporters such as P-glycoprotein (Pgp-*ABCB1*), breast cancer related protein (BCRP-*ABCG2*) and multiresistance protein (MRP-*ABCC1*) [10,11]. Despite the identification and knowledge of ABC transporters, the resulting pathways involved in MDR rise in IM resistance, causing leukemic cells to become resistant to therapy remain uncharacterized. Proteomic analyses by both 2-DE and MS-based methods have been widely used in comparative studies of protein expression patterns in cells or organisms in attempts to identify proteins involved in diverse maladies, including different types of cancer [12-18]. In an attempt to identify other proteins that could be associated with an IM therapy response through a MDR phenomenon in CML, we compared the proteomic profiles of the K562 (an erythroleukemic) cell line and the Lucena (K562/VCR - vincristine) cell line in our study. Initially, we showed that Lucena cells exhibit cross-resistance to IM. Than, using 2-DE and MALDI-TOF-TOF MS, we identified 36 differentially expressed proteins between these two cell lines. *In silico *characterization of the identified proteins showed that detoxification pathways and cellular maintenance *biofunctions *were increased in the resistant cell line. We selected over-expressed genes in resistant cells for quantitative validation in healthy donors and CML patients with different responses to IM therapy. The *leucine-rich PPR motif-containing (LRPPRC) protein, minichromosome maintenance complex component 7 (MCM7) *and *ATP-binding cassette sub-family B (MDR/TAP) member 1 (ABCB1) *genes were able to differentiate CML patients as either resistant or sensitive to IM therapy. Through the data presented in this work, we show that MDR is closely associated with resistance to IM and demonstrate its importance as a prognostic indicator for CML patients. Moreover, this proteomic approach identified LRPPRC and MCM7 as possible new targets associated with IM resistance.

## Results

### Differential IM response in CML cell lines

**Figure 1:**
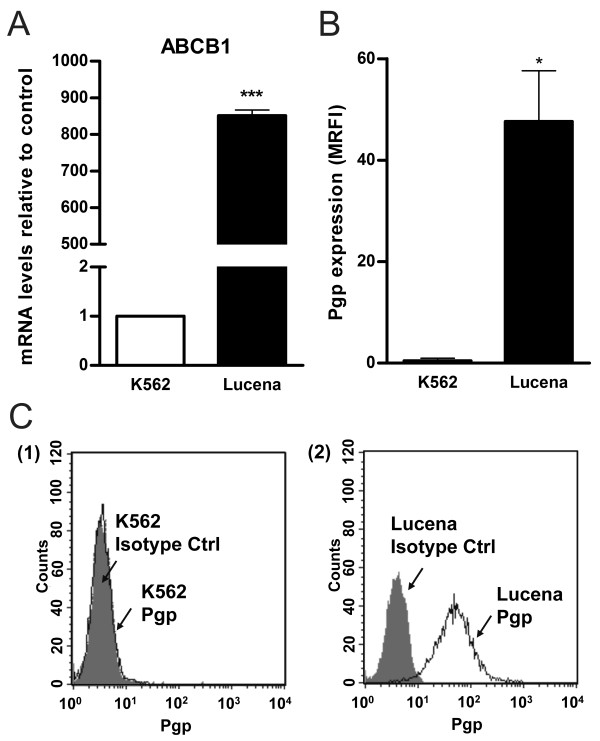
***ABCB1*/Pgp expression levels in K562 and Lucena cells**. **(A) RT-qPCR analysis of *ABCB1 *mRNA levels**. Raw expression values were normalized to β-actin expression. (**B**) Pgp expression by flow cytometry, represented as MRFI. (**C**) Representative histograms of Pgp expression. (1): K562 cells and (2): Lucena cells. PE-isotype antibody was used as control. Values represent the means of three independent determinations ± s.d (*p < 0.05)

**Figure 2:**
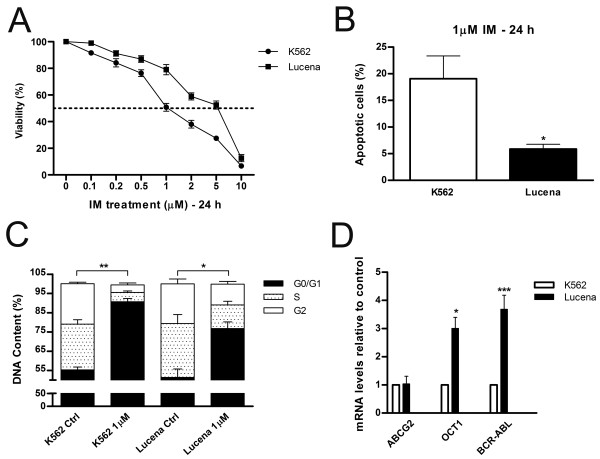
**Panel of Lucena cross-resistance to IM treatment**. (**A**) K562 and Lucena cell lines were treated with a range of IM doses for 24 h. Cell viability was measured by trypan blue exclusion assay. Apoptotic cells (**B**) and cell cycle (**C**) were measured by flow cytometry after 1 μM of IM treatment in both cell lines. (**D**) *ABCG2, OCT1 *and *BCR-ABL *mRNA expression levels in K562 and Lucena cell lines were quantified by RT-qPCR. Expression values were normalized to β-actin expression. Values represent the means of three independent determinations ± s.d. (*p < 0.05; **p < 0.01; ***p < 0.001)

**Figure 3:**
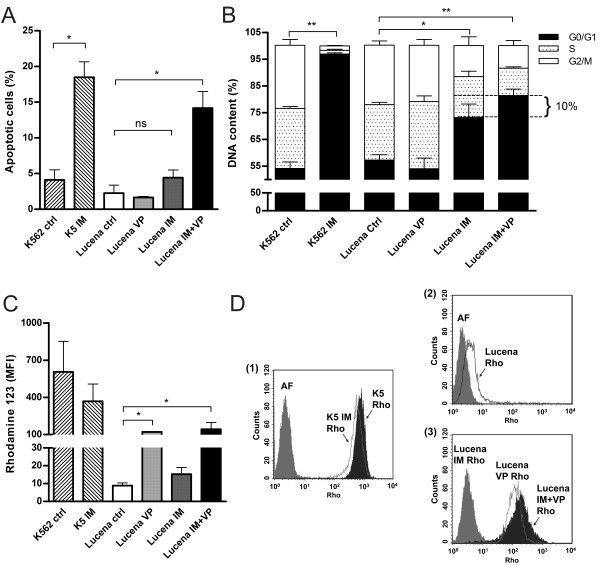
**Lucena cells cross-resistance to IM is due Pgp efflux**. Apoptotic cells (**A**), cell cycle (**B**) and Rho 123 (**C**-**D**) were measured by flow cytometry after 3 different treatments conditions: 1 μM IM, 50 μM VP and co-treatment with 1 μM IM and 50 μM VP. (**D**) Representative histograms of Rho 123 extrusion under conditions described above. (1): K562 ctrl and IM treatment; (2): Lucena ctrl and (3) Lucena under: IM, VP and IM + VP treatments. K562 cells were used as positive control for Rho 123 retention, and K562 treated with 1 μM IM was used as positive control for apoptosis induction and cell cycle arrest. Values represent the means of three independent determinations ± s.d. (*p < 0.05; **p < 0.01). AF = auto fluorescence; K5 = K562; LU = Lucena.

Lucena cells were established from K562 cells as a multidrug resistance lineage by vincristine (VCR) selection and their pattern of resistance includes a range of chemotherapy drugs [19]. Real time quantitative PCR (RT-qPCR) analysis of K562 and Lucena cell lines showed an 800.0-fold increase of *ABCB1 *mRNA levels by Lucena cells compared to K562 cells (Figure 1A). Over-expression of Pgp protein level was examined by flow cytometry and we observed a 45.0-fold increase expression of Pgp in Lucena cells compared to parental cells (Figure 1B-C). As demonstrated by Assef and colleagues, K562 cells managed to exhibit a MDR phenotype through VCR treatment also presented cross-resistance to IM [20]. Because the development of MDR cell lines is not well described in the literature (i.e., different concentrations of chemotherapy drugs are used) and VCR maintenance concentrations differ among these cell lines, we investigated IM effectiveness in Lucena cells. We treated both cell lines with different concentrations of the drug for 24 h and assayed cell viability. A comparative analysis of the viability of K562 and Lucena cells after IM treatment with 0.1 μM, 0.2 μM, 0.5 μM, 1 μM, 2 μM, 5 μM and 10 μM doses showed that Lucena cells were more resistant to IM than K562 cells (inhibitory concentration - IC_50 _5 μM and 1 μM, respectively; Figure 2A). To prove that the different cell viabilities were due to differential apoptotic activation between the cellular lineages, we conducted Annexin V assays with both cell lines under IM treatment with 1 μM (K562 IC_50 _dose). The results (Figure 2B) revealed that an IM dose of 1 μM activates apoptosis in approximately 20% of K562 cells but in only approximately 5% of Lucena cells. This result indicates that a 1 μM dose of IM for 24 h is insufficient to induce apoptosis in Lucena cells (Figure 2C) as it does in K562 cells. Moreover, cell cycle assay showed that IM treatment induced arrest in G0/G1 in both cell lines. However, this effect was more pronounced in K562 cells compared to Lucena cells (35.42% and 25.35%, respectively). Furthermore, we investigated the mRNA levels of *BCR-ABL *and the most related drug transporters involved in cancer with the goal of identifying the cause of Lucena cells cross-resistance to IM. In addition to possessing more *ABCB1*/Pgp than K562 cells (Figure 1), Lucena cells also had more *BCR-ABL *and *OCT1 *mRNA (Figure 2D), but did not presented significant difference in *ABCG2 *mRNA levels, which indicates that the failure of IM to induce apoptosis in Lucena cells may be due to Pgp drug efflux and/or by *BCR-ABL *up-regulation. In other to verify that cross-resistance to IM by Lucena cells might be through drug efflux, we performed apoptosis, cell cycle and Pgp activity assays on Lucena cells treated in 3 different conditions: 1 μM IM, 50 μM Verapamil (VP - Pgp blocker) and co-treated with 1 μM IM and 50 μM VP (Figure 3). VP treatment was not toxic and did not induce apoptosis (Figure 3A) and cell cycle arrest (Figure 3B) compared to control (untreated cells). Co-treatment enhanced cell cycle arrest in 10%, compared to IM alone (Figure 3B), which was followed by a 3.0-fold increase (approximately) in apoptosis compared to IM alone (Figure 3A). Rhodamine 123 (Rho 123) retention confirmed that K562 cells did not present functional Pgp efflux pump, even after IM treatment (Figure 3C-D). This accumulation was not verified in Lucena cells in both control (untreated cells) and IM treatment (Figure 3C-D), demonstrating that Pgp pump was functional in these cells. After VP treatment, irrespective of IM presence, Pgp was blocked, showing that Rho 123 was able to accumulate in these cells (Figure 3C-D).

### Identification of 2-DE differentially expressed proteins

**Figure 4:**
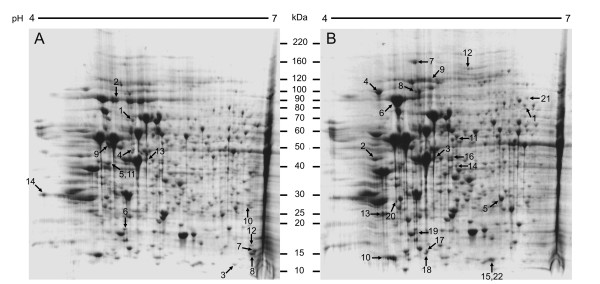
**Proteome maps of K562 (A) and Lucena (B) cell lines**. Nine hundred micrograms of total protein extract were separated by electrophoresis on IPG (pH 4-7) and gradient (8-18%) SDS-PAGE gels. 2-DE gels were stained with coomassie colloidal blue (CBB). The migration of molecular mass markers is represented in the middle. Numbers refer to the spot identity used in table **1**. Arrows correspond to the differentially expressed proteins according to ImageMaster 2D Platinum software.

Because the Lucena cell line has a cross-resistance to IM, we sought to generate Lucena and K562 cell lines (without IM treatment) proteomic profiles to understand the differences that contribute to IM cross-resistance. 2-DE was performed using 900 μg of total protein extract from each cell line. The 2-DE gels had more than 80% similarity, and 294 and 295 protein spots were visualized in the K562 and Lucena samples, respectively (Figure 4). All protein spots were excised from the 2-DE gels, digested with trypsin and analyzed by MALDI-TOF-TOF MS. Four hundred seventy-seven proteins were identified using this approach (235 proteins in K562 and 242 proteins in Lucena), which resulted in an identification rate of more than 80%. After an ImageMaster comparative analysis, only spots showing greater than 2.0 fold between samples (resistant versus responsive) were considered differentially expressed. In this analysis 36 proteins were found to be differentially expressed, with 14 proteins down-expressed and 22 proteins over-expressed in Lucena cells. Quantitative differences were observed, and proteins were analyzed and separated into cellular classes according to their potential biological function by gene ontology (GO) analyses http://www.geneontology.org (Table 1). Information regarding 2-DE analysis can be found in Additional file 1.

**Table 1:** Proteome map of differentially expressed proteins in K562 and Lucena cell lines.

Spot N°	Identified Protein	Fold Change > 2 **(Ratio Lucena vs. K562) **^***a***^
	***Structural Proteins***	

	*INCREASE*	

1 L	MSN Moesin	2.23

2 L	RPSA 33 kDa protein	3.45

3 L	ACTB Actin, cytoplasmic 1	2.75

	*DECREASE*	

1 K	LCP1 Plastin-2	- 3.65

	***Stress Response/Chaperone***	

	*INCREASE*	

4 L	HSP90B1 Endoplasmin precursor	2.22

5 L	HSPB1 Heat shock protein beta-1	4.23

6 L	HSP90AB1 Heat shock protein HSP 90-beta	3.1

7 L	HYOU1 Hypoxia up-regulated protein 1 precursor	3.76

8 L	VCP Transitional endoplasmic reticulum ATPase	2.29

	*DECREASE*	

2 K	HSP90AB1 Heat shock protein HSP 90-beta	- 4.17

	***Nucleic Acid Binding, Synthesis, Stability***	

	*INCREASE*	

9 L	AARS Alanyl-tRNA synthetase, cytoplasmic	3.43

10 L	RPA3 Replication protein A 14 kDa subunit	2.82

11 L	RBM17 Splicing factor 45	2.54

12 L	LRPPRC Leucine-rich PPR motif-containing protein, mitochondrial precursor	3.18

	*DECREASE*	

3 K	LSM2 U6 snRNA-associated Sm-like protein LSm2	- 3.23

4 K	HNRNPF Heterogeneous nuclear ribonucleoprotein F	- 3.01

5 K	HNRNPC Isoform C1 of Heterogeneous nuclear ribonucleoproteins C1/C2	- 3.22

	***Protein Binding and Synthesis***	

	*INCREASE*	

13 L	EIF3K Eukaryotic translation initiation factor 3 subunit K	2.96

	*DECREASE*	

6 K	EIF1AY Eukaryotic translation initiation factor 1A, Y-chromosomal	- 2.48

7 K	RPS12 ribosomal protein S12	-3.74

8 K	HINT1 Histidine triad nucleotide-binding protein 1	-3.57

	***Metabolism***	

	*INCREASE*	

14 L	ARG2 Arginase-2, mitochondrial precursor	3.83

15 L	COX6B1 Cytochrome c oxidase subunit VIb isoform 1	3.15

16 L	CKB Creatine kinase B-type	2.1

	*DECREASE*	

9 K	ATP5B ATP synthase subunit beta, mitochondrial precursor	- 2.83

10 K	TPI1 Isoform 1 of Triosephosphate isomerase	- 3.96

	***Signaling Transduction***	

	*INCREASE*	

17 L	SH3BGRL SH3 domain-binding glutamic acid-rich-like protein	3.04

18 L	TXNDC17 Thioredoxin domain-containing protein 17	2.68

19 L	GMFB GMFB protein	2.89

20 L	CAPNS1 Calpain small subunit 1	2.37

	*DECREASE*	

11 K	STRAP Serine-threonine kinase receptor-associated protein	- 3.22

	***Cell cycle/Proliferation***	

	*INCREASE*	

21 L	MCM7 Isoform 1 of DNA replication licensing factor MCM7	4.02

22 L	S100A11 Protein S100-A11	3.15

	***Unknown***	

	*DECREASE*	

12 K	C19orf10 UPF0556 protein C19orf10 precursor	- 3.12

13 K	MTPN Myotrophin	- 2.98

14 K	C1QBP Complement component 1 Q subcomponent-binding protein, mitochondrial	- 2.37

*^a ^*Fold change > 2 in resistance: cell lines have constitutively increased or decreased at least 2.0-fold changes of the given protein. Values expressed by ratio mean: Lucena/K562 (resistance versus responsive)

The corresponding spots on 2DE gels were identified with MS/MS

### *In silico *analysis of identified proteins

**Figure 5:**
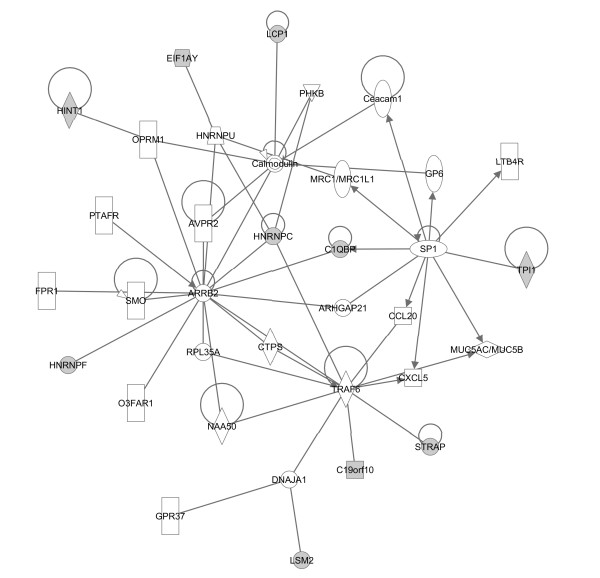
**Network analysis of down-expressed proteins involved in resistance**. The biological network was generated after the protein's dataset was uploaded into IPA. Gray nodes denote uploaded proteins, and white nodes denote proteins from the IPA database. Lines between the nodes indicate direct protein-protein interactions. Arrowheads show the direction of interaction. Self-regulation is indicated by lines that begin and end on the same node.

**Figure 6:**
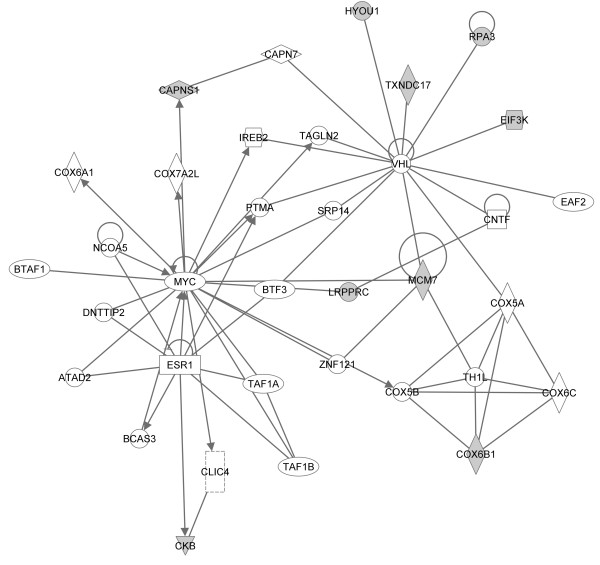
**Network analysis of over-expressed proteins involved in resistance**. The biological network was generated after the protein's dataset was uploaded into IPA. Gray nodes denote uploaded proteins, and white nodes denote proteins from the IPA database. Lines between the nodes indicate direct protein-protein interactions. Arrowheads show the direction of interaction. Self-regulation is indicated by lines that begin and end on the same node.

**Figure 7:**
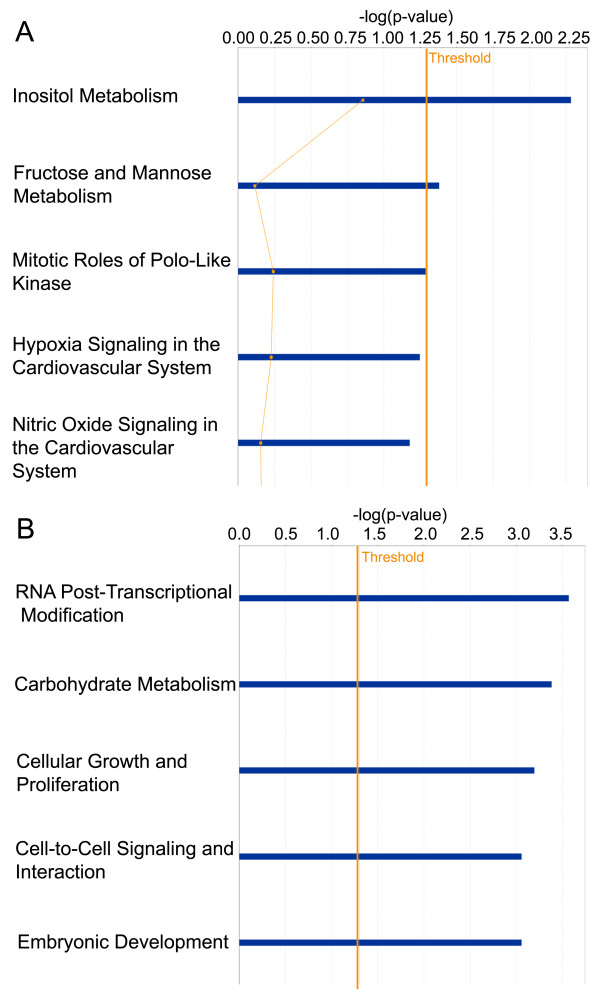
**IPA analysis of proteins down-expressed in resistance**. (**A**) Canonical Pathways analysis. The top 5 canonical pathways, are shown as determined by IPA. The *y-axis *shows the negative log of the *p-*value. (**B**) *Biofunction* analysis. The top 5 *biofunctions *among "Diseases and Disorders", "Molecular and Cellular Functions" and "Physiological System Development and Function" are shown as determined by IPA. The *y-axis *shows the negative log of the *p-*value.

**Figure 8:**
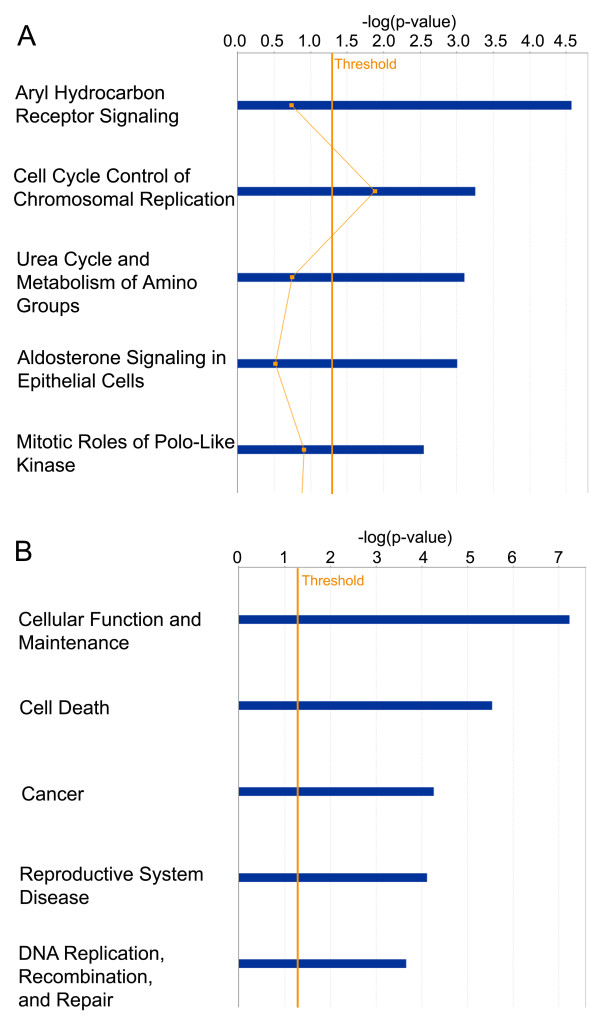
**IPA analysis of proteins over-expressed in resistance**. (**A**) Canonical Pathways analysis. The top 5 canonical pathways, are shown as determined by IPA. The *y*-*axis *shows the negative log of the *p*-value. (**B**) *Biofunction *analysis. The top 5 *biofunctions *among "Diseases and Disorders", "Molecular and Cellular Functions" and "Physiological System Development and Function" are shown as determined by IPA. The *y-axis *shows the negative log of the *p*-value.

To better understand resistance biology and to select the most promising candidates for further investigation, we assessed proteins using Ingenuity Pathway Analysis (IPA) (Ingenuity^® ^Systems, http://www.ingenuity.com). We only analyzed direct interactions of increased and decreased proteins (Lucena vs. K562) separately and compared these analyses. The identified proteins were then clustered into two major networks (Figures 5, 6), as well as broader *biofunctional *groups and canonical pathways, by IPA (Figures 7, 8). The created networks indicated *Cellular Function and Maintenance *(*p = *5.77E-8 - 3.97E-02, 8 molecules); *Cell Death *(*p = *2.86E-06 - 4.55E-02, 14 molecules); *DNA Replication, Recombination, and Repair *(*p = *2.18E-04 - 3.84E-02, 5 molecules); *Cell-to-Cell Signaling and Interaction *(*p = *1.29E-03 - 3.34E-02, 3 molecules) and *Small Molecule Biochemistry *(*p = *1.29E-03 - 3.34E-02, 7 molecules) as the most relevant molecular and cellular functions increased in resistance. Proteins identified by our proteomic approach are shown in gray, and they are sometimes present in more than one *biofunctional *group. The down-expressed protein dataset did not provide as many statistical results on predominant canonical pathways (Figure 7A) as the up-expressed protein dataset. However, it is known that the *Fructose and Mannose Metabolism *pathway is down-regulated in the MDR phenotype [21]. *Aryl Hydrocarbon Receptor (AHR) Signaling *(*p = *2.68E-05), *Cell Cycle Control of Chromosomal Replication *(p = 5.57E-04), *Urea Cycle and Metabolism of Amino Groups (p = *7.85E-04), *Aldosterone Signaling in Epithelial Cells (p = *9.86E-04) and *Mitotic Roles of Polo-Like Kinase (p = *2.83E-03), were the top five canonical pathways represented by the over-expressed proteins in resistant cells. *NRF2-mediated Oxidative Stress Response *(*p = *2.25E-04) and *Aryl Hydrocarbon Receptor (AHR) Signaling *(p = 4.57E-05) were considered the top pathways in the Toxicity List, also assessed by IPA analysis.

### Validation of target genes by real-time quantitative PCR

**Figure 9:**
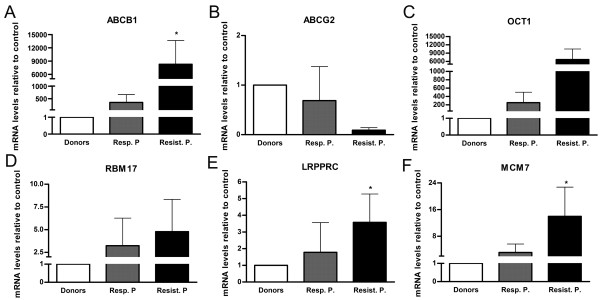
**Real-time quantitative PCR analysis of target gene expression in healthy donors and CML patients**. Total RNA was isolated from bone marrow donors and CML patients and examined by RT-qPCR to determine changes in mRNA levels. Raw expression values were normalized to β-actin expression. Analyses of *ABCB1, ABCG2, OCT1, RBM17, LRPPRC *and *MCM7 *expression changes were performed in 6 donors, 5 IM-responsive patients and 9 IM-resistant patients. Values represent the means of three independent determinations ± s.d. (*p < 0.05). Resp. P = responsive patients; Resist. P. = resistant patients.

Across a variety of possible candidates for validation, we selected *LRPPRC, MCM7 *and *RBM17 *as representative genes involved in the most representative molecular functions identified by IPA. This validation approach was selected due to limited amounts of patient samples. RT-qPCR methodology is a FDA-approved assay for clinics. RT-qPCR analysis was carried out to evaluate mRNA levels in cell lines (data not shown), healthy donors, IM-responsive patients and IM-resistant patients. Additionally, the expression of drug transporters such as *ABCB1, ABCG2 *and *OCT1 *was analyzed. Figure 9 shows their relative mRNA levels after normalization to *β-actin*. Analyses of drug transporters showed a significant over-expression of the *ABCB1 *in resistant patients. All genes selected from the proteomic approach were transcriptionally over-expressed in CML patients. After statistical analyses, only *RBM17 *did not show a significant difference in mRNA expression levels between healthy donors and IM-resistant CML patients.

### Identifying IM resistance targets by multivariate analyses

To determine if the expression of the drug transporters and target genes found by the proteomic approach, along with other variables, could indicate a response to IM therapy, we performed univariate and multivariate analyses with 14 CML patients (5 responsive and 9 resistant to IM therapy). We considered the following variables: target genes verified by RT-qPCR, molecular and cytogenetic response, disease phase (chronic, accelerated and blastic phases are denoted CP, AP and BP, respectively) and duration of disease. We constructed a receiver operating characteristic (ROC) curve to establish the cut-off point for each gene in order to categorize all mRNA expression levels found by RT-qPCR as either under or above these cut-off points. Using multivariate analysis, we calculated the Expβ for each variable, which is how much of an increase above basal level is necessary to increase the effect of each gene associated with all the genes studied. Because the increases of *ABCB1, LRPPRC *and *MCM7 *above their basal levels were statistically significant (Table 2), our analyses suggested these genes as important variables when analyzing IM therapy response. Their ROC curves can be found in the additional files data (see Additional file 2). Taken together, expression of these genes may correlate with response to IM therapy.

**Table 2:** Multivariate analyses of IM therapy failure.

Genes	ExpB	95% CI	P *^a^*	P_2 _*^b^*
ABCB1	18.865	0.83 - 425.88	0.041	
	
LRPPRC	2.867E-10	1.170E-11- 7.027E-9	0.022	0.013
	
MCM7	6.897E9	6.897E9- 6.897E9	0.005	

Abbreviation: ExpB, Exponential β; 95% CI, 95% confidence interval

*^a ^*p < 0.05 was considered to be significant

*^b ^*P_2_: Significance of all 3 genes together

Among the evaluated variables in multivariate analyses, only the target genes revealed by 2-DE, showed statistical significance in define CML patient's therapy status

## Discussion

Although the molecular basis of BCR-ABL-dependent mechanisms in IM resistance are well established (such as BCR-ABL mutations and *BCR-ABL *amplification), the same is not true for the BCR-ABL-independent mechanisms. The complexity of BCR-ABL independent resistance has not led to targeted therapy development. Instead, current approaches are focused on overcoming resistance of the T315I mutation, targeting survival pathways, and multi-kinase inhibitors [22]. Various cellular mechanisms may be involved in the nature of cellular resistance. Cells exposed to toxic compounds can develop resistance by a number of mechanisms including increased amount of drug target, inhibition of apoptosis, changes in gene expression that control cell cycle, enhanced DNA repair, decreased drug uptake, or increased detoxification [23]. Baran and colleagues have pointed some important insights on IM resistance mediated by anti-apoptotic signals. In IM-resistant cells developed by their group, over-expression of Bcl-2 (anti-apoptotic gene) led to mitochondrial membrane potential (MMP) increase [24]. Besides, they examined the role of Sphingosine kinase-1 (SK-1)/sphingosine 1-phosphate (SP1) signaling in IM resistance. SK-1/SP1 activation can promote resistance of IM-induced apoptosis through unbalance between the levels of C_18_-ceramide (pro-apoptotic) and SP1 (anti-apoptotic) [25]. Recently, they determined a novel mechanism in which SK1/SP1 mediates BCR-ABL1 stability and drug resistance by protein phosphatase 2A modulation [26]. Constitutive activation of downstream BCR-ABL signaling molecules such as STAT3, STAT5A, Lyn, NF-kB, ERK1/2, and AKT are also considered as independent mechanisms of IM resistance. These molecules have been studied as potential targets for overcoming resistance [27-29]. Mencalha and colleagues demonstrated that LLL3, a STAT3 inhibitor, led to a decrease in proliferation and viability of BCR-ABL positive cells. As so, LLL3 administered together with IM increased the sub-G1 DNA content by 25% compared to IM treatment alone, demonstrating an additive effect of IM-induced apoptosis [30]. Regarding on CML stem cells, it is a consensus in literature that these cells are intrinsically resistant to TK inhibition [31-35]. Interesting, Hamilton and colleagues have shown recently in transgenic mouse model of CML-like disease and in derived CML stem cells from patients, that CML stem cell survival is BCR-ABL kinase independent. They suggest that curative approaches in CML must focus on kinase-independent mechanisms of resistance [36].

Despite these novel information regarding BCR-ABL independent mechanisms, MDR phenotype still remains poorly understood. Little is known about the mechanisms through which MDR is activated, the molecules that are being induced, the pathways that are altered and exactly which proteins contribute to IM resistance. It is possible that the MDR phenotype functions in the same way for all drugs, but it is also possible that specific accessory proteins play important roles in resistance to distinct and unrelated drugs.

Recently, some groups have demonstrated that K562 cells driven to acquire the MDR phenotype also presented cross-resistance to IM compared to their parental cell lines [37]. This phenomenon is not exclusive by VCR selection in vitro. Illmer and colleagues [38] observed, in a model of K562-VP16 (etoposide) cells, a gradually increasing in Pgp expression with subsequent decline of intracellular IM levels. Decreased IM levels were associated with a retained phosphorylation pattern of the BCR-ABL target CRKL and loss of effect of IM on cellular proliferation and apoptosis. Yamada and colleagues [39] showed similar results related to IM effectiveness in K562-ADM (adriamycin) cells over-expressing Pgp. Pgp over-expression impact on cellular concentration of IM (and others TK inhibitors) was shown by Haouala and colleagues by knocking-down Pgp and measuring intra/extracellular IM ratio [40]. According to Vasconcelos and colleagues [41], an IM treatment at different concentrations for 24 h in K562 and Lucena cells was sufficient to increase *ABCB1 *at mRNA levels and Pgp protein levels only in Lucena cells. Based on these data, we ought to investigate IM effect on cellular viability, apoptosis and cell cycle. As showed in Figure 2A, after 24 h of IM treatment, Lucena cells presented an IC_50 _of 5 μM, which is 5.0-fold greater than K562 cells' IC_50_. These findings corroborate with Vasconcelos and colleagues' observation that Lucena cells over-express Pgp at 5 μM, compared to control (untreated cells). This variation of Pgp could explain Lucena cells' viability under IM treatment and the delay in apoptosis and cell cycle arrest in G0/G1, compared to K562 cells (Figure 2B-C). Moreover, our results summarized in Figure 3 provide evidence that blockage of Pgp pump, due to VP treatment, increased apoptosis and cell cycle arrest induced by IM in Lucena cells, demonstrating that cross-resistance to IM may be, among others mechanisms, through Pgp drug efflux.

In order to identify proteins involved in BCR-ABL-independent mechanisms through MDR, we used this model in a comparative proteomic study between CML K562 and Lucena (K562/VCR) cell lines. Proteomic approaches such as 2-DE and MS enable the identification of differentially expressed proteins. In addition, these studies also provide pictures of alterations, making possible to better comprehend the biology and mechanisms that lead to a cellular process under investigation.

Our proteomic study of K562 and Lucena cells identified 36 differentially expressed proteins. The diversity of the proteins confirms that several pathways are deregulated and act together to promote the development of resistance. Among the pathways identified by IPA analyses as increased in resistance, we highlight the NF-E2-related factor 2 (NRF2)-mediated oxidative stress response and aryl hydrocarbon receptor (AHR) signaling. The key transcription factors of these pathways are Nrf2 and AHR, respectively. Through their translocation into the nucleus and binding to their respective elements in targets genes, they promote up-regulation of antioxidant and detoxification genes, stress response genes, xenobiotic-metabolizing genes, genes involving the ubiquitin-mediated proteassomal degradation system, intracellular redox-regulating genes, genes controlling cell growth and genes encoding transporters, such as ABC family members [42,43]. The relationship between these pathways and chemoresistance in several types of cancer is well known [44-46]. However, only recently has NRF2 signaling been under investigation for its association with BCR-ABL^+ ^cell IM resistance. Ozawa and co-workers demonstrated that up-regulation of NRF2 conferred IM resistance to KCL22 cells through transcription of the γ-GCS light subunit (γ-GCSl), a major determinant for glutathione (GSH) homeostasis [47]. The role of GSH in IM resistance was also discussed by Colavita and colleagues in another proteomic study [48]. This same group also showed that NRF2 up-regulation by HEME increased the IM IC_50 _without changing BCR-ABL kinase activity. NRF2 repression restored IM sensitivity. These results were verified by Bonovolias and Tsiftsoglou in a study with CML and acute myeloid leukemia BCR-ABL^+ ^cells [49,50]. Thus, these findings highlight the importance of investigating not only NRF2 but also the role of AHR signaling in IM resistance with the aim of gaining additional knowledge on the mechanistic basis of IM resistance.

The separate evaluation of networks allowed us to observe that the increased proteins found due to resistance by our approach are localized in the center of the network. This result indicates that these proteins are either in contact with the cytoskeletal proteins or are involved with cellular proliferation/maintenance proteins as key connecting proteins. It is clear that these interactions can lead to cellular maintenance through cytoskeletal changes that may confer alterations in cellular organization, cell cycle and proliferation. These alterations may give an adaptive advantage to resistant cells. *LRPPRC, MCM7 *and *RBM17 *were chosen as representative genes involved in these cellular processes. Quantitative results permitted the verification of a tendency of increased expression of target gene mRNA levels in IM-resistant patients compared to IM-responsive patients. Multivariate analyses sorted *ABCB1, LRPPRC *and *MCM7 *as statistically significant genes in IM therapy status.

LRPPRC is an approximately 130 kDa protein that was first identified as over-expressed in the human liver carcinoma cell line HepG2 [51]. This protein seems to be involved in several intracellular processes such as homeostasis and microtubule alteration [52,53]. It seems that LRPPRC may be related to transactivation of MDR genes (*ABCB1 *and *LRP*) by invMED sequence in acute lymphoid leukemia [54]. Moreover, an anti-apoptotic role for LRPPRC has been recently determined in hepatocarcinoma cells [55]. In up-regulated network created by IPA, LRPPRC interacts directly with BTF3 protein, which forms a stable complex with RNA polymerase II and is involved with regulation of transcription initiation. BTF3 interacts with MYC protein, which has a well-established role in cell proliferation and CML evolution. Along, these information support the potential role of LRPPRC in cell maintenance, proliferation, regulation of transcription and apoptosis.

MCM7 protein belongs to a highly conserved group of proteins that are essential for the initiation of DNA replication and cell proliferation [56]. This protein can be regulated by MYCN (v-myc myelocytomatosis viral related oncogene, neuroblastoma derived - avian) transcription factor in neuroblastoma [57]. Additionally, there is evidence that *ABCB1 *can also be regulated by MYCN in neuroblastoma [58,59]. This information is relevant as we found that MCM7 was over-expressed IM-resistant patients. MYCN could be regulating both genes in CML, so overexpression of MCM7 could be indirectly related to resistance. Alternatively, MCM7 may be directly involved in resistance by altering DNA repair function, as shown previously [60-62]. In IPA network MCM7 was shown to interact with TLH1 and ZNF121 proteins, which are involved in regulation of transcriptional activity. As LRPPRC, MCM7 is also implicated with resistance *biofunctions *found in our study such as cellular maintenance, cell cycle control and cell proliferation.

The involvement of *ABCB1 *in IM resistance is controversial, but our results demonstrate that the *ABCB1 *gene was significantly over-expressed in IM-resistant patients. The 9 IM-resistant patients in our study did not have mutations at the ABL site, and 5 patients were in the CP or AP stage. This result indicates that CML patients in the BC stage are not the only individuals that over-express this gene, as has been suggested in the literature [63,64]. Ferrao and colleagues have shown that the *ABCB1 *gene does not confer IM resistance in vitro [65], but several studies have provided contradictory results. Specifically, studies with patients have shown that over-expression or polymorphisms of the *ABCB1 *gene can alter the response to therapy [66-72]. For this reason, a hypothesis was derived that tried to explain the relationship between *ABCB1 *over-expression and IM resistance. This hypothesis was based on the fact that *ABCB1 *is expressed in early primitive normal hematopoietic stem cells [73,74] and can be over-expressed in leukemic stem cells, which would aid in maintenance of leukemia [75]. However, evidence shows that silencing *ABCB1 *in leukemic stem cells does not sensitize them to IM treatment in culture [76,77]. The role of the *ABCB1 *gene in IM resistance remains unclear. However, our findings, along with published data, suggest that *ABCB1 *could be considered a prognostic factor for CML, as it is in acute myeloid leukemia.

Identification of potentially useful proteomic-based biomarkers must be validated in larger, well-defined retrospective and prospective clinical studies, and these combined efforts should result in identification of biomarkers that will greatly improve early detection, prognosis and prediction of treatment response [78].

In conclusion, our comparative proteomic approach using CML MDR/IM cross-resistant cell line and its parental cell line identified LRPPRC and MCM7 as putative actors in IM resistance. These data were validated in healthy donors and CML patients with different therapy responses. Altogether the expression of these genes and *ABCB1 *could discriminate responsive and resistant groups and the therapy state of patients. As we analyzed a small patient cohort, we sought validation by future prospective clinical studies to establish biomarker's application in treatment response prediction or in follow-up monitoring.

## Methods

### Culture conditions

Lucena (a K562 multidrug-resistant cell line induced by VCR) cells over-expressing the *ABCB1 *gene were kindly provided by Dr. Vivian Rumjanek (Departamento de Bioquímica Médica, Universidade Federal do Rio de Janeiro, Brazil). The human myelogenous leukemia cell line (K562) and its VCR-resistant derivative Lucena were grown in RPMI 1640 medium supplemented with 10% fetal bovine serum FBS, 50 units/mL penicillin G, 50 μg/L streptomycin and 2 mM L-glutamine (all from Invitrogen) at 37°C in a humidified atmosphere containing 5% CO_2_. Lucena medium was supplemented with 60 nM VCR (Sigma).

### Imatinib mesylate treatment

Cell lines were exposed to different doses of IM dissolved in DMSO (Sigma-Aldrich) with final concentration of 0.5%. DMSO-treated cells were used as vehicle-controls. Treatments were carried out in 12-well culture plates for a period of 24 h with a cell density of 2.0 × 10^5 ^cells/mL. IM concentrations of 0.1 μM, 0.2 μM, 0.5 μM, 1 μM, 2 μM, 5 μM and 10 μM were used for cell viability assays. For apoptosis and cell cycle assays, a 1 μM IM dose was used. Cells were treated with 1 μM IM, 50 μM VP [79] and co-treated with 1 μM IM and 50 μM VP for apoptosis, cell cycle and Pgp activity assays. K562 cells were used as positive control for Rho 123 retention and K562 cells treated with 1 μM IM were used as positive control for apoptosis induction and cell cycle arrest. All experiments were performed in triplicate.

### Pgp expression assay

To determine Pgp expression, we analyzed both cell lines with anti-Pgp-PE (phycoerythrin) antibody (Beckman Coulter) according to manufacturer's instructions. Briefly, after treatment, 5.0 × 10^5 ^cells were harvested, washed twice with cold PBS, resuspended in 1 mL PBS/BSA (0.2% Azide, 1% BSA) and incubated for 15 min. After incubation, cells were harvested and anti-Pgp-PE (5 μL) was added, and the sample was incubated for 30 min in the dark. After incubation, 2 mL of PBS/BSA were added to each sample. Cells were harveshed and resuspended in PBS/1%Formol. For every condition, 20.000 events were acquired using a FACSCalibur Flow Cytometer (Becton Dickinson, USA) and analyzed using CellQuest v.3.1 Software (Becton Dickinson, USA). Results are expressed as mean relative fluorescence intensity (MRFI), which was calculated by subtracting the mean fluorescence intensity (MFI) for specific antibody by the MFI of the respective, isotype control. All experiments were performed in triplicate.

### Cell viability assay

Aliquots of IM-treated cells were removed after 24 h of treatment. The number of viable cells was determined using the trypan blue exclusion assay. The concentration of drug necessary to achieve a 50% reduction of viable cells was denoted as the IC_50_. All experiments were performed in triplicate.

### Apoptosis assays

To determine the percentage of apoptotic cells, we analyzed phosphatidyl serine externalization and membrane integrity by double staining with Annexin V PE and 7-AAD (PE Annexin V Apoptosis Detection Kit I, BD Pharmingen, USA) according to manufacturer's instructions. Briefly, after treatment, 1.0 × 10^5 ^cells were harvested, washed twice with cold PBS and resuspended in 100 μL of 1× binding buffer. Annexin V PE (5 μL) and 7-AAD (5 μL) were added, and samples were incubated for 15 min in the dark. After incubation, 400 μL of 1× binding buffer was added to each sample. Cells positive for Annexin V PE and 7-AAD were considered apoptotic. For every condition, 20.000 events were acquired using a FACSCalibur Flow Cytometer (Becton Dickinson, USA) and analyzed using CellQuest v.3.1 Software (Becton Dickinson, USA). All experiments were performed in triplicate.

### Cell cycle assays

Cell cycle was evaluated by staining with propidium iodide (PI, Sigma-Aldrich) [80]. Approximately 3.0 × 10^5 ^cells were resuspended in 400 μL of hypotonic buffer (3.4 mM Tris-HCl (pH 7.6), 10 mM NaCl, 0.1% (v/v) NP-40, 700 U/L RNase, and 0.075 mM PI) and incubated for 30 min at 4°C. For every condition, 5.000 events were acquired in a FACSCalibur Flow Cytometer (Becton Dickinson, USA) and analyzed using Cell Quest v.3.1 Software (Becton Dickinson, USA). All experiments were performed in triplicate.

### Pgp activity assay

Rho 123 (Sigma) was used to measure the activity of Pgp by flow cytometry [81]. For each experiment, 1.0 × 10^5^cells were incubated with 200 ng/mL of Rho 123. After 30 min of incubation at 37°C/5% CO_2 _cells were washed with PBS and analyzed in a FACSCalibur Flow Cytometer (Becton Dickinson, USA). For every condition, 20.000 events were acquired using a FACSCalibur Flow Cytometer (Becton Dickinson, USA) and analyzed using CellQuest v.3.1 Software (Becton Dickinson, USA). Results are expressed as MFI. All experiments were performed in triplicate.

### Bone marrow samples

All bone marrow samples were obtained from CML patients in all disease phases (CB, AP, or BP) and donors admitted or registered at the Instituto Nacional de Câncer (Rio de Janeiro, Brazil), according to the guidelines of the local Ethics Committee and the Helsinki declaration. We selected 6 healthy donors (mean age = 30, range = 20-37, male:female ratio = 4:2), 5 IM-responsive patients (mean age = 45, range = 33-52, male:female ratio = 5:0, CP:AP:BP ratio = 3:2:0) and 9 IM-resistant patients (mean age = 44, range = 26-61, male:female ratio = 5:4, CP:AP:BP ratio = 1:4:4). Diagnoses and follow-ups were based on hematologic, cytogenetic and molecular assays. IM-responsive patients exhibited a major molecular response and complete hematologic and cytogenetic response, whereas IM-resistant patients lacked hematologic, cytogenetic and molecular responses. The inclusion criterion was to investigate CML patients that received IM as a first-line therapy. Marrow aspirates were collected in heparinized tubes and processed on the day they were collected. Bone marrow mononuclear cells were isolated from 2-5 mL of aspirate in a Ficoll-Hypaque density gradient (Ficoll 1.077 g/mL; GE, Sweden) according to manufacturer's protocol. Cells were washed 3 times in PBS and subsequently used for RNA extraction.

### 2-DE

Cells were washed in PBS and resuspended in cold lysis buffer containing 50 mM Tris (pH 7.5), 5 mM EDTA, 10 mM EGTA, 50 mM NaF, 20 mM β-glycerophosphate, 250 mM NaCl, 2% NP-40 and protease inhibitors and incubated on ice for 30 min. The lysates were centrifuged at 12.000 *g *for 15 min at 4°C. The supernatants were collected, and the total protein concentrations were determined by the Bradford assay [82]. Nine hundred micrograms of total cell protein were precipitated using a 2D cleanup kit (GE, Sweden) according to manufacturer's instructions and resuspended in buffer containing 6 M Urea, 2 M Thiourea, 15 mM DTT, 2% (w/v) ASB14, 0.5% IPG buffer (pH 3-10; GE, Sweden) and bromophenol blue traces. IEF was carried out in an 11-cm Immobiline DryStrip (pH 4-7; GE, Sweden) on an Ettan IPGphor III electrophoresis unit (GE, Sweden) for a total of 32070 Vh. Subsequently, IPG strips were equilibrated for 15 min in equilibration buffer (6 M Urea, 30% (w/v) Glycerol, and 2% SDS in 0.05 M Tris-HCl (pH 8.8) containing 100 mg DTT per 10 mL SDS equilibration buffer) and then equilibrated for 15 min in buffer containing 250 mg iodoacetamide. IPG strips were run on ExcelGel SDS 8-18% gels according to procedures recommended by the manufacturer (GE, Sweden) and stained with colloidal blue [83]. All gels were scanned with an Image scanner using LabScan v.5.0 software (GE, Sweden), and ImageMaster 2D Platinum v.6.0 software (GE, Sweden) and subjected to visual analysis. p*I *values were determined using a linear 4-7 distribution using a logarithmic curve. Molecular weight values were determined using a Benchmark protein standard (Invitrogen). 2-DE gels were analyzed separately and averaged. For spots found in all gels, the normalized spot volumes of triplicate samples were averaged. Spot normalization is an internal calibration that makes the data independent of experimental variations between gels caused by conditions such as differences in protein loading or staining. It was performed with the use of relative Volume (%Vol) to quantify and compare the gel spots. The intensity of each spot was quantified by calculating the spot volume after normalization of the image using the total spot volume normalization method multiplied by the total area of all the spots.

### MS identification

The protein spots of interest were cut out of the gel and processed for MS according to the following protocol. The trimmed gels were washed three times in 50% acetonitrile (AcN) and 25 mM NH_4 _HCO_3 _(pH 8.0) for 15 min, soaked in 100% AcN and dried in a Speed-Vac for 30 min. The samples were rehydrated at 4°C in digestion buffer (25 mM NH_4 _HCO_3 _(pH 8.0) containing 15 ng/μL porcine trypsin (Sequencing Grade Modified Trypsin, Promega, USA)) and incubated at 37°C overnight. The peptides were extracted with a solution of 50% AcN and 5% Trifluoroacetic acid (TFA) and dried in a Speed-Vac. Peptides were redissolved in 3 μL of 50% AcN/1% TFA solution. For MALDI MS/MS analysis, 0.5 μL of the redissolved peptide was mixed with fresh cyano hydroxycinnamic acid as a matrix on a MALDI plate. Mass spectra for peptide mass fingerprinting and confirmatory fragmentation analysis were acquired using the MALDI-TOF-TOF instrument 4700 (Applied Biosystems, USA). MS data were acquired in MALDI ion source, in positive ion reflector mode, mass range 900-4000 Da, using a neodymium-doped yttrium aluminum garnet (Nd: YAG) laser with a 200-Hz repetition rate and collision-induced dissociation (CID) mode *off*. Capillary electrophoresis (CE) was not used. Typically, 1.600 shots were accumulated for spectra in MS mode while 2.400 shots were accumulated for spectra in MS/MS mode. Up to eight of the most intense ion signal was selected as precursors for MS/MS acquisition. Spectra were acquired after plate calibration with calibration mixture 1 or 2 (Sequazyme Peptide Mass Standards kit, PerSeptive Biosystems, USA).

### Data processing and bioinformatics analysis

Peak lists were generated by Data Explorer v.4.5 software (Applied Biosystems, USA) using default parameters and searched with Mascot Daemon v.2.1 software (Matrix Science) against the non-redundant International Protein Index (IPI) protein sequence database v.3.6 (80,412 entries, released 17.06.09). Search parameters were as follows: database searches were restricted to Homo sapiens, precursor ion and fragment ion mass tolerance of 0.2 Da, tryptic specificity allowing for one missed cleavage, fixed modification of carbamidomethylation of cysteine residues, and variable modification of oxidation of methionine residues. The criterion for positive protein identification was a minimum of two peptides. The Mascot threshold (relied on a 5% probability that the protein identification is incorrect) is a probability score. This score is described in Supplemental Figure 1 for each protein identified. The data associated with this manuscript may be downloaded from the http://ProteomeCommons.org Tranche network using the following hash:

F6rVqTaM9oCMmblj4oHeNBhczim01D4sJYEw5AurFjuk2n0WgLexO4PUOeJpB7RmFDDaPX + bbIxKBe1Vj7dbHgHjjX0AAAAAAACMpQ = =

These data include all MS files (.t2d format) from differentially expressed proteins identified. Proteins were investigated according to their GO http://www.geneontology.com annotations based on molecular functions. Functional analyses, network constructions and canonical pathway analyses were generated through the use of IPA (Ingenuity^® ^Systems, http://www.ingenuity.com). The following parameters were used: reference set (Ingenuity knowledge Base - genes only); relationships to consider (direct relationships); network generation (mode *on*, 35 molecules per network); data sources (all); confidence (consider only relationships that were experimentally observed OR high predicted); species (human); tissues and cell lines (all) and mutations (all).

### Real-time quantitative PCR analysis

Analysis of mRNA levels was carried out by RT-qPCR. Two micrograms of TRIzol- (Invitrogen) extracted RNA from cell lines and healthy donor- and patient-derived mononuclear bone marrow cells were reverse transcribed with Superscript II Reverse Transcriptase^® ^(Invitrogen). cDNA dilutions (1:100) were mixed with SYBR Green PCR Master Mix^® ^(Applied Biosystems, USA) and the following forward (Fow) and reverse (Rev) primers: *ABCB1 *NM_000927.3 Fow 5' CCCATCATTGCAATAGCAGG 3', Rev 5' GTTCAAACTTCTGCTCCTGA 3'; *ABCG2 *NM_004827.2 Fow 5' TGGCTGTCATGGCTTCAGTA 3', Rev 5' GCCACGTGATTCTTCCACAA 3'; *OCT1 *NM_003057.2 Fow 5' TCCTCTTCCTGCTCTACTACT 3', Rev 5' ATGAAGGGCTCAGCTTTTCGG 3'; *LRPPRC *NM_133259.3 Fow 5' GAGAGATGCCGGAATTGAGC 3', Rev 5' CTCGGACTTCTCCACCTTCT 3'; *MCM7 *NM_005916.3 Fow 5' TCGAGGCATGAAAATCCG GG 3', Rev 5' CGCCAGTCGATCAATGTATGACA 3'; *RBM17 *NM_032905.4 Fow 5' GTGGGTTTGCAAGGAGACCAG3', Rev 5' AAGTGGGTGGGGCAATGG 3'; *ACTB *NM_001101.3 Fow 5' ACCTGAGAACTCCACTACCCT 3', Rev 5' GGTCCCACCCATGTTCCAG 3'. RT-qPCR was performed in a Rotor Gene 6000 thermocycler (Cobertt) with 50 cycles of 20 s at 95°C, 30 s at 60°C and 30 s at 72°C. For each sample, the expression of target genes was normalized to *β-actin *mRNA levels. Changing mRNA levels were evaluated as previously described [84].

### Statistical analysis

Cell viability, apoptosis activation, DNA content/cell cycle and mRNA level differences between K562 and Lucena were compared by a paired *t*-test. These statistical analyses were performed using GraphPad Prism^® ^v.5 software (GraphPad, USA). To determine if increased mRNA levels were associated with IM resistance, cut-off points for each gene were selected. These cut-offs were determined by constructing ROC curves with estimations of sensitivity, specificity and area under the curve. Clinical information on IM resistance was used as the state variable. The cut-off point for each gene was established, and all values were categorized under or above these points. For univariate analyses, Chi-squared and Fisher's exact tests were used to analyze the association between mRNA levels and IM treatment outcome. For multivariate analyses, logistic regression was performed to determine the association between mRNA levels and IM therapy outcome. These statistics were performed using SPSS v.13.0 for Windows^® ^software (SPSS Inc.). *P*- Values less than 0.05 were considered as statistically significant (*p < 0.05, **p < 0.01, and ***p < 0.001).

## List of Abbreviations

CML: chronic myeloid leukemia; TK: tyrosine kinase; IM: imatinib mesylate; MDR: multidrug resistance; ABC: ATP-binding cassette; VCR: vincristine; RT-qPCR: real-time quantitative PCR; IC^50^: inhibitory concentration; VP: verapamil; Rho123: rhodamine 123; GO: Gene Ontology; IPA: Ingenuity Pathway Analysis; FDA: Food and Drug Administration; CP: chronic phase; AP: accelerated phase; BP: blastic phase; ROC: receiver operating characteristic; VP16: etoposide; ADM: Adriamycin; MRFI: mean relative fluorescence intensity; MFI: mean fluorescence intensity; PE: phycoerythrin; PI: propidium iodide; AcN: Acetonitrile; TFA: Trifluoroacetic acid; Nd:YAG: neodymium-doped yttrium aluminum garnet; CID: collision-induced dissociation; CE: capillary electrophoresis; IPI: International Protein Index; Fow: forward; Rev: reverse.

## Competing interests

The authors declare that they have no competing interests.

## Authors' contributions

SC performed the experiments, bioinformatics analysis and drafted the manuscript. LP participated in the design of the study and mass spectrometry protein identification. BR participated in flow cytometry experiments. AM assisted the real-time quantitative PCR experiments. DP participated in statistical analysis. EA participated in the design of this study. All authors read and approved the final manuscript.

## Supplementary Material

Additional file 1 **Differentially expressed proteins identified in IM cross-resistance**. Proteins were separated into biological functions according to GO analysis. Information regarding pI/MW, statistical and Mascot score, number of identified peptides, peptide sequence and sequence coverage were described. Click here for file

Additional file 2 **ROC curve**. ROC curve analysis showed that *ABCB1*. *LRPPRC *and *MCM7 *genes, together, are potential candidate biomarkers for IM therapy response for further investigation. Area under curve (AUC): 0.733 (ABCB1); 0.550 (LRPPRC); 0.767 (MCM7). Click here for file

## References

[B1] Jiang X Zhao Y Smith C Gasparetto M Turhan A Eaves A Eaves C Chronic myeloid leukemia stem cells possess multiple unique features of resistance to BCR-ABL targeted therapies Leukemia 2007 21 926 935 17330101 10.1038/sj.leu.2404609

[B2] Hochhaus A Chronic myelogenous leukemia (CML): resistance to tyrosine kinase inhibitors Ann Oncol 2006 17 274 279 10.1093/annonc/mdl273 17018738

[B3] Mughal TI Goldman JM Emerging strategies for the treatment of mutant Bcr-Abl T315I myeloid leukemia Clin Lymphoma Myeloma 2007 7 S81 S84 10.3816/CLM.2007.s.006 17382017

[B4] Quintás-Cardama A Kantarjian HM Cortes JE Mechanisms of Primary and Secondary Resistance to Imatinib in Chronic Myeloid Leukemia Cancer Control 2009 16 122 131 19337198 10.1177/107327480901600204

[B5] Walz C Sattler M Novel targeted therapies to overcome imatinib mesylate resistance in chronic myeloid leukemia Crit Rev Oncol Hematol 2006 57 145 164 10.1016/j.critrevonc.2005.06.007 16213151

[B6] Swords R Alvarado Y Giles F Novel Abl kinase inhibitors in chronic myeloid leukemia in blastic phase and Philadelphia chromosome-positive acute lymphoblastic leukemia Clin Lymphoma Myeloma 2007 7 S113 S119 10.3816/CLM.2007.s.011 17382020

[B7] Azzariti A Porcelli L Simone GM Quatrale AE Colabufo NA Berardi F Perrone R Zucchetti M D'Incalci M Xu JM Paradiso A Tyrosine kinase inhibitors and multidrug resistance proteins: interactions and biological consequences Cancer Chemother Pharmacol 2009 65 335 346 19495754 10.1007/s00280-009-1039-0

[B8] Mahon F Hayette S Lagarde V Belloc F Turcq B Nicolini F Belanger C Manley P Leroy C Etienne G Roche S Pasquet J Evidence that resistance to nilotinib may be due to BCR-ABL, Pgp, or Src kinase overexpression Cancer Res 2008 68 9809 9816 10.1158/0008-5472.CAN-08-1008 19047160

[B9] Dohse M Scharenberg C Shukla S Robey RW Volkmann T Deeken JF Brendel C Ambudkar SV Neubauer A Bates SE Comparison of ATP-binding cassette transporter interactions with the tyrosine kinase inhibitors imatinib, nilotinib, and dasatinib Drug Metab Dispos 2010 38 1371 1380 10.1124/dmd.109.031302 20423956 PMC2913625

[B10] Lage H An overview of cancer multidrug resistance: a still unsolved problem Cell Mol Life Sci 2008 65 3145 3167 10.1007/s00018-008-8111-5 18581055 PMC11131739

[B11] Stavrovskaya AA Stromskaya TP Transport proteins of the ABC family and multidrug resistance of tumor cells Biochemistry (Mosc) 2008 73 592 604 10.1134/S0006297908050118 18605983

[B12] Cristea IM Gaskell SJ Whetton AD Proteomics techniques and their application to hematology Blood 2004 103 3624 3634 10.1182/blood-2003-09-3295 14726377

[B13] Cho WC Contribution of oncoproteomics to cancer biomarker discovery Molecular Cancer 2007 2 6 25 10.1186/1476-4598-6-25 PMC1852117 17407558

[B14] Pizzatti L Sá LA De Souza JM Bisch PM Abdelhay E Altered protein profile in chronic myeloid leukemia chronic phase identified by a comparative proteomic study Biochim Biophys Acta 2006 1764 929 942 16581319 10.1016/j.bbapap.2006.02.004

[B15] Chuthapisith S Layfield R Kerr ID Hughes C Eremin O Proteomic profiling of MCF-7 breast cancer cells with chemoresistance to different types of anti-cancer drugs Int J Oncol 2007 30 1545 1551 17487377

[B16] Zeindl-Eberhart E Haraida S Liebmann S Jungblut PR Lamer S Mayer D Jäger G Chung S Rabes HM Detection and identification of tumor-associated protein variants in human hepatocellular carcinomas Hepatology 2004 39 540 549 10.1002/hep.20060 14768008

[B17] An HJ Kim DS Park YK Kim SK Choi YP Kang S Ding B Cho NH Comparative proteomics of ovarian epithelial tumors J Proteome Res 2006 5 1082 1090 10.1021/pr050461p 16674097

[B18] Yang YX Hu HD Zhang DZ Ren H Identification of Proteins Responsible for the Development of Adriamycin Resistance in Human Gastric Cancer Cells Using Comparative Proteomics Analysis J Biochem Mol Biol 2007 40 853 860 10.5483/BMBRep.2007.40.6.853 18047778

[B19] Rumjanek VM Trindade GS Wagner-Souza K de-Oliveira MC, Marques-Santos LF, Maia RC, Capella MA: Multidrug resistance in tumour cells: characterization of the multidrug resistant cell line K562-Lucena 1 An Acad Bras Cienc 2001 73 57 69 10.1590/S0001-37652001000100007 11246270

[B20] Assef Y Rubio F Coló G del Mónaco S Costas MA Kotsias BA Imatinib resistance in multidrug-resistant K562 human leukemic cells Leuk Res 2009 33 710 716 10.1016/j.leukres.2008.09.024 18977528

[B21] Wang X Lu Y Yang J Shi Y Lan M Liu Z Zhai H Fan D Identification of triosephosphate isomerase as an anti-drug resistance agent in human gastric cancer cells using functional proteomic analysis J Cancer Res Clin Oncol 2008 134 995 1003 10.1007/s00432-008-0367-5 18309519 PMC12160761

[B22] Roychowdhury S Talpaz M Managing resistance in chronic myeloid leukemia Blood Reviews 2011 25 279 290 10.1016/j.blre.2011.09.001 21982419

[B23] Brózik A Hegedüs C Erdei Z Hegedüs T Özvegy-Laczka C Szakács G Sarkadi B Tyrosine kinase inhibitors as modulators of ATP binding cassette multidrug transporters: substrates, chemosensitizers or inducers of acquired multidrug resistance? Expert Opin Drug Metab Toxicol 2011 7 623 642 10.1517/17425255.2011.562892 21410427

[B24] Baran Y Ural AU Gunduz U Mechanisms of cellular resistance to imatinib in human chronic myeloid leukemia cells Hematology 2007 12 497 503 10.1080/10245330701384179 17852433

[B25] Baran Y Salas A Senkal CE Gunduz U Bielawski J Obeid LM Alterations of Ceramide/Sphingosine 1-Phosphate Rheostat Involved in the Regulation of Resistance to Imatinib-induced Apoptosis in K562 Human Chronic Myeloid Leukemia Cells J Biol Chem 2007 282 15 10922 10934 10.1074/jbc.M610157200 17303574

[B26] Salas A Ponnusamy S Senkal CE Meyers-Needham M Selvam SP Saddoughi SA Apohan E Sentelle RD Smith C Gault CR Obeid LM El-Shewy HM Oaks J Santhanam R Marcucci G Baran Y Mahajan S Fernandes D Stuart R Perrotti D Ogretmen B Sphingosine kinase-1 and sphingosine 1-phosphate receptor 2 mediate Bcr-Abl1 stability and drug resistance by modulation of protein phosphatase 2A Blood 2011 117 22 5941 5952 10.1182/blood-2010-08-300772 21527515 PMC3112039

[B27] Bewry NN Nair RR Emmons MF Boulware D Pinilla-Ibarz J Hazlehurst LA Stat3 contributes to resistance toward BCR-ABL inhibitors in a bone marrow microenvironment model of drug resistance Mol Cancer Ther 2008 7 3169 3175 10.1158/1535-7163.MCT-08-0314 18852120 PMC2676735

[B28] Kosova B Tezcanli B Ekiz HA Cakir Z Selvi N Dalmizrak A Kartal M Gunduz U Baran Y Suppression of STAT5A increases chemotherapeutic sensitivity in imatinib-resistant and imatinib-sensitive K562 cells Leuk Lymphoma 2010 51 1895 1901 10.3109/10428194.2010.507830 20849385

[B29] Nambu T Araki N Nakagawa A Kuniyasu A Kawaguchi T Hamada A Saito H Contribution of BCR-ABL-independent activation of ERK1/2 to acquired imatinib resistance in K562 chronic myeloid leukemia cells Cancer Sci 2010 10 137 142 19843070 10.1111/j.1349-7006.2009.01365.x PMC11158207

[B30] Mencalha AL Du Rocher B Sales D Binato R Abdelhay E LLL-3, a STAT3 inhibitor, represses BCR-ABL-positive cell proliferation, activates apoptosis and improves the effects of Imatinib mesylate Cancer Chemother Pharmacol 2010 65 1039 1046 10.1007/s00280-009-1109-3 19701750

[B31] Graham SM Jorgensen HG Allan E Pearson C Alcorn MJ Richmond L Holyoake TL Primitive, quiescent, Philadelphia-positive stem cells from patients with chronic myeloid leukemia are insensitive to STI571 in vitro Blood 2002 99 319 325 10.1182/blood.V99.1.319 11756187

[B32] Bhatia R Holtz M Niu N Gray R Snyder DS Sawyers CL Arber DA Slovak ML Forman SJ Persistence of malignant hematopoietic progenitors in chronic myelogenous leukemia patients incomplete cytogenetic remission following imatinib mesylate treatment Blood 2003 101 4701 4707 10.1182/blood-2002-09-2780 12576334

[B33] Angstreich GR Matsui W Huff CA Vala MS Barber J Hawkins AL Griffin CA Smith BD Jones RJ Effects of imatinib and interferon on primitive chronic myeloid leukaemia progenitors Br J Haematol 2005 130 373 381 10.1111/j.1365-2141.2005.05606.x 16042686

[B34] Copland M Hamilton A Elrick LJ Baird JW Allan EK Jordanides N Barow M Mountford JC Holyoake TL Dasatinib (BMS-354825) targets an earlier progenitor population than imatinib in primary CML but does not eliminate the quiescent fraction Blood 2006 107 4532 4539 10.1182/blood-2005-07-2947 16469872

[B35] Nicholson E Holyoake T The chronic myeloid leukemia stem cell Clin Lymphoma Myeloma 2009 9 S376 S381 10.3816/CLM.2009.s.037 20007106

[B36] Hamilton A Helgason GV Schemionek M Zhang B Myssina S Allan EK Nicolini FE Müller-Tidow C Bhatia R Brunton VG Koschmieder S Holyoake TL Chronic myeloid leukemia stem cells are not dependent on Bcr-Abl kinase activity for their survival Blood 2011 doi: 10.1182/blood-2010-12-326843 10.1182/blood-2010-12-326843 PMC3286213 22184410

[B37] Mukai M Che XF Furukawa T Sumizawa T Aoki S Ren XQ Haraguchi M Sugimoto Y Kobayashi M Takamatsu H Akiyama S Reversal of the resistance to STI571 in human chronic myelogenous leukemia K562 cells Cancer Sci 2003 94 557 563 10.1111/j.1349-7006.2003.tb01482.x 12824882 PMC11160154

[B38] Illmer T Schaich M Platzbecker U Freiberg-Richter J Oelschlägel U von Bonin M Pursche S Bergemann T Ehninger G Schleyer E P-glycoprotein-mediated drug efflux is a resistance mechanism of chronic myelogenous leukemia cells to treatment with imatinib mesylate Leukemia 2004 18 401 408 10.1038/sj.leu.2403257 14724652

[B39] Yamada O Ozaki K Furukawa T Machida M Wang Y Motoji T Mitsuishi T Akiyama M Yamada H Activation of STAT5 confers imatinib resistance on leukemic cells through the transcription of *TER *and *MDR* Cell Signal 2011 23 1119 1127 10.1016/j.cellsig.2011.02.005 21356308

[B40] Haouala A Widmer N Duchosal MA Montemurro M Buclin T Decosterd LA Drug interactions with the tyrosine kinase inhibitors imatinib, dasatinib, and nilotinib Blood 2011 117 e75 87 10.1182/blood-2010-07-294330 20810928

[B41] Vasconcelos F Silva K Souza P Silva L Moellmann-Coelho A Klumb C Maia R Variation of MDR Proteins Expression and Activity Levels According to Clinical Status and Evolution of CML Patients Cytometry B Clin Cytom 2011 80 158 166 21520403 10.1002/cyto.b.20580

[B42] Wang XJ Sun Z Villeneuve NF Zhang S Zhao F Li Y Chen W Yi X Zheng W Wondrak GT Wong PK Zhang DD Nrf2 enhances resistance of cancer cells to chemotherapeutic drugs, the dark side of Nrf2 Carcinogenesis 2008 29 1235 1243 10.1093/carcin/bgn095 18413364 PMC3312612

[B43] Ma Q Xenobiotic-activated receptors: from transcription to drug metabolism to disease Chem Res Toxicol 2008 21 1651 1671 10.1021/tx800156s 18707139

[B44] Hayashibara T Yamada Y Mori N Harasawa H Sugahara K Miyanishi T Kamihira S Tomonaga M Possible involvement of aryl hydrocarbon receptor (AhR) in adult T-cell leukemia (ATL) leukemogenesis: constitutive activation of AhR in ATL Biochem Biophys Res Commun 2003 300 128 134 10.1016/S0006-291X(02)02793-6 12480531

[B45] Kizu R Okamura K Toriba A Kakishima H Mizokami A Burnstein KL Hayakawa K A role of aryl hydrocarbon receptor in the antiandrogenic effects of polycyclic aromatic hydrocarbons in LNCaP human prostate carcinoma cells Arch Toxicol 2003 77 335 343 12799773 10.1007/s00204-003-0454-y

[B46] Giudice A C. Arra C, M.C. Turco MC: Review of molecular mechanisms involved in the activation of the Nrf2-ARE signaling pathway by chemopreventive agents Methods Mol Biol 2010 647 37 74 10.1007/978-1-60761-738-9_3 20694660

[B47] Tarumoto T Nagai T Ohmine K Miyoshi T Nakamura M Kondo T Mitsugi K Nakano S Muroi K Komatsu N Ozawa K Ascorbic acid restores sensitivity to imatinib via suppression of Nrf2-dependent gene expression in the imatinib-resistant cell line Exp Hematol 2004 32 375 381 10.1016/j.exphem.2004.01.007 15050748

[B48] Colavita I Esposito N Martinelli R Catanzano F Melo JV Pane F Ruoppolo M Salvatore F Gaining insights into the Bcr-Abl activity-independent mechanisms of resistance to imatinib mesylate in KCL22 cells: A comparative proteomic approach Biochim Biophys Acta 2010 1804 1974 1987 20417730 10.1016/j.bbapap.2010.04.009

[B49] Nagai T Kikuchi S Ohmine K Miyoshi T Nakamura M Kondo T Furuyama K Komatsu N Ozawa K Hemin reduces cellular sensitivity to imatinib and anthracyclins via Nrf2 J Cell Biochem 2008 104 680 691 10.1002/jcb.21659 18172853

[B50] Bonovolias ID Tsiftsoglou AS Hemin counteracts the repression of Bcl-2 and NrF2 genes and the cell killing induced by imatinib in human Bcr-Abl(+) CML cells Oncol Res 2009 17 535 547 10.3727/096504009789745557 19806784

[B51] Hou J Wang F McKeehan WL Molecular cloning and expression of the gene for a major leucine-rich protein from human hepatoblastoma cells (HepG2) In Vitro Cell Dev Biol Anim 1994 30 111 114 10.1007/BF02631402 8012652

[B52] Liu L McKeehan WL Sequence analysis of LRPPRC and its SEC1 domain interaction partners suggests roles in cytoskeletal organization, vesicular trafficking, nucleocytosolic shuttling, and chromosome activity Genomics 2002 79 124 136 10.1006/geno.2001.6679 11827465 PMC3241999

[B53] Liu L Amy V Liu G McKeehan WL Novel complex integrating mitochondria and the microtubular cytoskeleton with chromosome remodeling and tumor suppressor RASSF1 deduced by in silico homology analysis, interaction cloning in yeast, and colocalization in cultured cells In Vitro Cell Dev Biol Anim 2002 38 582 594 10.1290/1543-706X(2002)38<582:NCIMAT>2.0.CO;2 12762840 PMC3225227

[B54] Labialle S Dayan G Gayet L Rigal D Gambrelle J Baggetto LG New invMED1 element cis-activates human multidrug-related MDR1 and MVP genes, involving the LRP130 protein Nucleic Acids Res 2004 32 3864 3876 10.1093/nar/gkh722 15272088 PMC506807

[B55] Michaud M Barakat S Magnard S Rigal D Baggetto LG Leucine-rich protein 130contributes to apoptosis resistance in human hepatocarcinoma cells Intern Journal of Oncol 2011 38 169 178 21109938

[B56] Takisawa H Mimura S Kubota Y Eukaryotic DNA replication: from pre-replication complex to initiation complex Curr Opin Cell Biol 2000 12 690 696 10.1016/S0955-0674(00)00153-8 11063933

[B57] Shohet JM Hicks MJ Plon SE Burlingame SM Stuart S Chen SY Brenner MK Nuchtern JG Minichromosome maintenance protein MCM7 is a direct target of the MYCN transcription factor in neuroblastoma Cancer Res 2002 62 1123 1128 11861392

[B58] Blanc E Goldschneider D Ferrandis E Barrois M Le Roux G Leonce S Douc-Rasy S Bénard J Raguénez G MYCN enhances P-gp/MDR1 gene expression in the human metastatic neuroblastoma IGR-N-91 model Am J Pathol 2003 163 321 331 10.1016/S0002-9440(10)63656-5 12819037 PMC1868150

[B59] Pajic M Norris MD Cohn SL Haber M The role of the multidrug resistance-associated protein 1 gene in neuroblastoma biology and clinical outcome Cancer Lett 2005 228 241 246 10.1016/j.canlet.2005.01.060 15979785

[B60] Tsao CC Geisen C Abraham RT Interaction between human MCM7 and Rad17 proteins is required for replication checkpoint signaling EMBO J 2004 23 4660 4669 10.1038/sj.emboj.7600463 15538388 PMC533049

[B61] Cortez D Glick G Elledge SJ: Minichromosome maintenance proteins are direct targets of the ATM and ATR checkpoint kinases Proc Natl Acad Sci USA 2004 101 10078 10083 10.1073/pnas.0403410101 15210935 PMC454167

[B62] Chou DM Elledge SJ Tipin and Timeless form a mutually protective complex required for genotoxic stress resistance and checkpoint function Proc Natl Acad Sci USA 2006 103 18143 18147 10.1073/pnas.0609251103 17116885 PMC1654129

[B63] Mahon FX Deininger M Schultheis B Chabrol J Reiffers J Goldman JM Melo JV Selection and characterization of BCR-ABL positive cell lines with differential sensitivity to the signal transduction inhibitor STI571: diverse mechanisms of resistance Blood 2000 96 1070 1079 10910924

[B64] Reis FR Vasconcelos FC Pereira DL Moellman-Coelho A Silva KL Maia RC Survivin and P-glycoprotein are associated and highly expressed in late phase chronic myeloid leukemia Oncol Rep 2011 26 471 478 21567097 10.3892/or.2011.1296

[B65] Ferrao PT Frost MJ Siah SP Ashman LK Overexpression of P-glycoprotein in K562 cells does not confer resistance to the growth inhibitory effects of imatinib (STI571) in vitro Blood 2003 102 4499 4503 10.1182/blood-2003-01-0083 12881321

[B66] Dai H Marbach P Lemaire M Hayes M Elmquist WF Distribution of STI-571 to the brain is limited by P-glycoprotein-mediated efflux J Pharmacol Exp Ther 2003 304 1085 1092 12604685 10.1124/jpet.102.045260

[B67] Mahon FX Belloc F Lagarde V Chollet C Moreau-Gaudry F Reiffers J Goldman JM Melo JV MDR1 gene overexpression confers resistance to imatinib mesylate in leukemia cell line models Blood 2003 101 2368 2373 10.1182/blood.V101.6.2368 12609962

[B68] Thomas J Wang L Clark RE Pirmohamed M Active transport of imatinib into and out of cells: implications for drug resistance Blood 2004 104 3739 3745 10.1182/blood-2003-12-4276 15315971

[B69] Galimberti S Cervetti G Guerrini F Testi R Pacini S Fazzi R Simi P Petrini M Quantitative molecular monitoring of BCR-ABL and MDR1 transcripts in patients with chronic myeloid leukemia during Imatinib treatment Cancer Genet Cytogenet 2005 162 57 62 10.1016/j.cancergencyto.2005.01.015 16157201

[B70] Ni LN Li JY Miao KR Qiao C Zhang SJ Qiu HR Qian SX Multidrug resistance gene (MDR1) polymorphisms correlate with imatinib response in chronic myeloid leukemia Med Oncol 2011 28 265 269 10.1007/s12032-010-9456-9 20204543

[B71] Yamakawa Y Hamada A Nakashima R Yuki M Hirayama C Kawaguchi T Saito H Association of genetic polymorphisms in the influx transporter SLCO1B3 and the efflux transporter ABCB1 with imatinib pharmacokinetics in patients with chronic myeloid leukemia Ther Drug Monit 2011 33 244 250 21311410 10.1097/FTD.0b013e31820beb02

[B72] Deenik W van der Holt B Janssen JJWM Chu IWT Valk PJM Ossenkoppele GJ van der Heiden IP P. Sonneveid P, van Schaik RHN, Cornelissen JJ: Polymorphisms in the multidrug resistance gene MDR1 (ABCB1) predict for molecular resistance in patients with newly diagnosed chronic myeloid leukemia receiving high-dose imatinib Blood 2010 116 6144 6145 10.1182/blood-2010-07-296954 21183698

[B73] Zhou S Schuetz JD Bunting KD Colapietro AM Sampath J Morris JJ Lagutina I Grosveld GC Osawa M Nakauchi H Sorrentino BP The ABC transporter Bcrp1/ABCG2 is expressed in a wide variety of stem cells and is a molecular determinant of the side-population phenotype Nat Med 2001 7 1028 1034 10.1038/nm0901-1028 11533706

[B74] Jordanides NE Jorgensen HG Holyoake TL Mountford JC Functional ABCG2 is overexpressed on primary CML CD34+ cells and is inhibited by imatinib mesylate Blood 2006 108 1370 1373 10.1182/blood-2006-02-003145 16627755

[B75] Jiang X Zhao Y Smith C Gasparetto M Turhan A Eaves A Eaves C Chronic myeloid leukemia stem cells possess multiple unique features of resistance to BCR-ABL targeted therapies Leukemia 2007 21 926 935 17330101 10.1038/sj.leu.2404609

[B76] Hatziieremia S Jordanides NE Holyoake TL Mountford JC Jørgensen HG Inhibition of MDR1 does not sensitize primitive chronic myeloid leukemia CD34+ cells to imatinib Exp Hematol 2009 37 692 700 10.1016/j.exphem.2009.02.006 19394750

[B77] Zong Y Zhou S Sorrentino BP Loss of P-glycoprotein expression in hematopoietic stem cells does not improve responses to imatinib in a murine model of chronic myelogenous leukemia Leukemia 2005 19 1590 1596 10.1038/sj.leu.2403853 16001089

[B78] Leth-Larsen R Lund RR Ditzel HJ Plasma membrane proteomics and its application in clinical cancer biomarker discovery Mol Cell Proteomics 2010 9 1369 1382 10.1074/mcp.R900006-MCP200 20382631 PMC2938092

[B79] Rocha GG Simões M Oliveira RR Kaplan MAC Gattass CR 3β-acetyl tormentic acid induces apoptosis of resistant leukemia cells independently of P-gp/ABCB1 activity or expression Invest New Drugs 2012 30 105 113 10.1007/s10637-010-9524-1 20814731

[B80] Nicoletti I Migliorati G Pagliaccil MC Grignani F Riccardi C A rapid and simple method for measuring thymocyte apoptosis by propidium iodide staining and flow cytometry J Immunol Methods 1991 139 271 279 10.1016/0022-1759(91)90198-O 1710634

[B81] Neyfakh AA Use of fluorescent dyes as molecular probes for the study of multidrug resistance Exp Cell Res 1988 174 168 176 10.1016/0014-4827(88)90152-8 3335222

[B82] Bradford MM A rapid and sensitive method for the quantitation of microgram quantities of protein utilizing the principle of protein-dye binding Anal Biochem 1976 72 248 254 10.1016/0003-2697(76)90527-3 942051

[B83] Candiano G Bruschi M Musante L Santucci L Ghiggeri GM Carnemolla B Orecchia P Zardi L Righetti PG Blue silver: a very sensitive colloidal Coomassie G-250 staining for proteome analysis Electrophoresis 2004 25 1327 1333 10.1002/elps.200305844 15174055

[B84] Livak KJ Schmittgen TD Analysis of relative gene expression data using real-time quantitative PCR and the 2(-Delta Delta C (T)) Method Methods 2001 25 402 408 10.1006/meth.2001.1262 11846609

