# Supplementary material for: A comparative proteomic study identified LRPPRC and MCM7 as putative actors in imatinib mesylate cross-resistance in Lucena cell line
Source: Proteome Sci. 2012 Mar 30;10:23. doi: 10.1186/1477-5956-10-23 (PMC3361502; doi:10.1186/1477-5956-10-23)
Supplement: Additional file 1 — Differentially expressed proteins identified in IM cross-resistance. Proteins were separated into biological functions according to GO analysis. Information regarding pI/MW, statistical and Mascot score, number of identified peptides, peptide sequence and sequence coverage were described. [file 1477-5956-10-23-S1.PDF]

Table 1. Differentially expressed proteins identified in IM cross-resistance. Proteins were separated into biological functions according to GO analysis. Information regarding pI/MW, statistical and Mascot score, number of identified peptides, peptide sequence and sequence coverage were described.

Identified Protein	Accession Number [a]	Theorical pI/MW	Experimental pI/MW	Statistical Score p<0.05	Mascot Score	N° of Unique Peptides	Peptides Sequence	Sequence Cov. (%) [b]
Structural Proteins								
INCREASE								
MSN Moesin	IPI00219365	6.08 / 67892	6.5 / 83488	33	70	3	R.IQVWHEEHR.G	4
							K.IGFPWSEIR.N	
							K.APDFVFYAPR.L	
RPSA 33 kDa protein	IPI00413108	4.79 / 33464	4.81 / 48765	32	255	6	K.FLAAGTHLGGTNLDFQMEQYIYK.R Oxidation (M)	36
							R.AIVAIENPADVSVISSR.N	
							K.FAAATGATPIAGR.F	
							R.FTPGTFTNQIQAAFR.E	
							R.ADHQPLTEASYVNLPTIALCNTDSPLR.Y	
							K.GAHSVGLMWWMLAR.E 2 Oxidation (M)	
ACTB Actin, cytoplasmic 1	IPI00021439	5.29 / 42052	5.41 / 46719	33	225	5	R.VAPEEHPVLLTEAPLNPK.A	18
							K.SYELPDGQVITIGNER.F	
							K.DLYANTVLSGGTTMYPGIADR.M	
							K.DLYANTVLSGGTTMYPGIADR.M Oxidation (M)	
							K.QEYDESGPSIVHR.K	
DECREASE								
LCP1 Plastin-2	IPI00010471	5.2 / 70815	5.37 / 75973	33	36	2	K.IGLFADIELSR.N	3
							K.LNLAFIANLFNR.Y	

Stress Response / Chaperone								
INCREASE								
HSP90B1 Endoplasmin precursor	IPI00027230	4.76 / 92696	4.87 / 116504	33	139	4	K.FAFQAEVNR.M	5
							K.SILFVPTSAPR.G	
							R.GLFDEYGSK.K	
							K.GVVDSDDLPLNVSR.E	
HSPB1 Heat shock protein beta-1	IPI00025512	5.98 / 22826	6.21 / 27565	33	142	3	R.GPSWDPFR.D	16
							R.LFDQAFGLPR.L	
							R.VSLDVNHFAPDELTVK.T	
HSP90AB1 Heat shock protein HSP 90-beta	IPI00414676	4.97 / 83554	5.05 / 99183	33	225	5	K.IDIIPNPQER.T	8
							K.HFSVEGQLEFR.A	
							R.RAPFDLFENK.K	
							R.GVVDSEDLPLNISR.E	
							K.HLEINPDHPIVETLR.Q	
HYOU1 Hypoxia up-regulated protein 1 precursor	IPI00000877	5.16 / 111494	5.28 / 176811	32	131	6	K.DAVITVPVFFNQAER.R	8
							K.VLQLINDNTATALSYGVFR.R	
							R.LAGLFNEQR.K	
							R.VEFEELCADLFER.V	
							R.DAVVYPILVEFTR.E	
							K.AHFNLDESGVLSLDR.V	
VCP Transitional endoplasmic reticulum ATPase	IPI00022774	5.14 / 89950	5.34 / 107671	32	140	7	K.VVETDPSPYCIVAPDTVIHCEGEPIKR.E	15
							R.EDEEESLNEVGYDDIGGCR.K	
							R.WALSQSNPSALR.E	
							R.ETVVEVPQVTWEDIGGLEDVKR.E	
							R.ELQELVQYPVEHPDK.F	
							K.GVLFYGPPGCGK.T	
							K.GPELLTMWFGESEANVR.E Oxidation (M)	
DECREASE								

HSP90AB1 Heat shock protein HSP 90-beta	IPI00414676	4.97 / 83554	5.32 / 99183	33	212	7	K.HNDDEQYAWESSAGGSFTVR.A	12
							K.EDQTEYLEER.R	
							K.HSQFIGYPITLYLEK.E	
							K.SLTNDWEDHLAVK.H	
							K.HFSVEGQLEFR.A	
							R.RAPFDLFENK.K	
							K.HLEINPDHPIVETLR.Q	
Nucleic Acid Binding, Synthesis, Stability								
INCREASE								
AARS Alanyl-tRNA synthetase, cytoplasmic	IPI00027442	5.34 / 107484	5.46 / 124738	33	76	2	K.VGDQVWLFIDEPR.R	2
							R.AVFDETYPDPVR.V	
RPA3 Replication protein A 14 kDa subunit	IPI00017373	4.96 / 13674	4.96 / 15250	33	54	2	K.EDSHPFDLGLYNEAVK.I	27
							K.IIHDFPQFYPLGIVQHD. -	
RBM17 Splicing factor 45	IPI00176706	5.76 / 45162	5.73 / 57718	33	99	3	R.DFPYEEDSRPR.S	3
							K.CVIFEIPGAPDDEAVR.I	
							R.IFLEFER.V	
LRPPRC Leucine-rich PPR motif-containing protein, mitochondrial precursor	IPI00783271	5.81 / 159003	5.86 / 156498	61	62	11	R.LQWFCDR.C	11
							K.FSPTDFLAK.M	
							K.ALYEHLTAK.N	
							K.AGYPQYVSEILEK.V	
							R.NLLESYHVPELIK.D	
							K.LGAVYDVSHYNALLK.V	
							R.AGDMENAENILTVMR.D	
							K.VIEPQYFGLAYLFR.K	
							K.DLPVTEAVFSALVTGHAR.A	
							K.EQNIVFNAETYSNLIK.L	
							K.TKDLPVTEAVFSALVTGHAR.A	
DECREASE								

LSM2 U6 snRNA-associated Sm-like protein LSm2	IPI00032460	6.04 / 10942	6.45 / 13810	32	154	2	K.NDLSICGTLHSVDQYLNIK.L<br>R.YVQLPADEVDTQLLQDAAR.K	48
HNRNPF Heterogeneous nuclear ribonucleoprotein F	IPI00003881	5.38 / 45985	5.49 / 51668	32	102	2	K.ITGEAFVQFASQELAEK.A<br>K.ATENDIYNFFSPLNPVR.V	8
HNRNPC Isoform C1 of Heterogeneous nuclear ribonucleoproteins C1/C2	IPI00216592	4.94 / 32375	5.11 / 44759	33	105	2	K.GFAFVQYVNER.N<br>R.VPPPPPIAR.A	6
Protein Binding and Synthesis								
INCREASE								
EIF3K Eukaryotic translation initiation factor 3 subunit K	IPI00033143	4.81 / 25329	4.92 / 25554	32	37	2	R.YNPENLATLER.Y<br>K.FICHVVGITYQHIDR.W	11
DECREASE								
EIF1AY Eukaryotic translation initiation factor 1A, Y-chromosomal	IPI00232533	5.07 / 16546	5.28 / 19934	33	142	3	K.KVWINTSDIILVGLR.D<br>K.VWINTSDIILVGLR.D<br>K.AYGELPEHAK.I	17
RPS12 ribosomal protein S12	IPI00847579	6.81 / 14905	6.67 / 15920	33	80	2	K.LVEALCAEHQINLIK.V<br>K.DVIEEYFK.C	17
HINT1 Histidine triad nucleotide-binding protein 1	IPI00239077	6.43 / 13907	6.64 / 15402	32	102	4	K.AQVARPGGDTIFGK.I<br>R.CLAFHDISPQAPTHFLVIPK.K<br>R.MVVNEGSDGGQSVYHVHLHVLGGR.Q<br>R.MVVNEGSDGGQSVYHVHLHVLGGR.Q Oxidation (M)	46
Metabolism								
INCREASE								
ARG2 Arginase-2, mitochondrial precursor	IPI00020332	6 / 38839	5.75 / 43188	32	37	3	K.DDLYNNLIVNPR.S<br>R.SVGLANQELAEVVSR.A<br>K.TTANLAVDVIASSFGQTR.E	12
COX6B1 Cytochrome c oxidase subunit VIb isoform 1	IPI00216085	6.54 / 10414	6.09 / 14087	33	100	4	R.FPNQNQTR.N<br>R.NCWQNYLDFHR.C<br>K.GGDISVCEWYQR.V	47

							R.VYQSLCPTSWVTDWDEQR.A	
CKB Creatine kinase B-type	IPI00022977	5.34 / 42902	5.68 / 46386	33	38	3	R.GFCLPPHCSR.G	10
							K.LAVEALSSLDGDLAGR.Y	
							K.TFLVWVNEEDHLR.V	
DECREASE								
ATP5B ATP synthase subunit beta, mitochondrial precursor	IPI00303476	5.26 / 56525	5.11 / 56085	33	493	9	R.LVLEVAQHLGESTVR.T	22
							K.IPVGPETLGR.I	
							K.AHGGYSVFAGVGER.T	
							K.VALVYGQMNEPPGAR.A	
							K.VALVYGQMNEPPGAR.A Oxidation (M)	
							R.VALTGLTVAEYFR.D	
							R.DQEGQDVLLFIDNIFR.F	
							R.AIAELGIYPAVDPLDSTSR.I	
							R.IMDPNIVGSEHYDVAR.G Oxidation (M)	
TPI1 Isoform 1 of Triosephosphate isomerase	IPI00465028	5.65 / 31057	6.6 / 27442	33	236	6	K.FFVGGNWK.M	34
							K.VPADTEVVCAPPTAYIDFAR.Q	
							K.DCGATWVVLGHSER.R	
							R.RHVFGESDELIGQK.V	
							K.VVLAYEPVWAIGTGK.T	
							K.ELASQPDVDGFLVGGASLKPEFVDIINAK.Q	
Signaling Transduction								
INCREASE								
SH3BGRL SH3 domain-binding glutamic acid-rich-like protein	IPI00025318	5.22 / 12766	5.43 / 15815	33	74	2	R.GDYDAFFEAR.E	24
							R.ENNAVYAFLGLTAPPGSK.E	
TXNDC17 Thioredoxin domain-containing protein 17	IPI00646689	5.4 / 14217	5.41 / 15607	33	46	3	R.YEEVSVSGFEEFHR.A	30
							K.TIFAYFTGSK.D	
							K.SWCPDCVQAEPVVR.E	
GMFB GMFB protein	IPI00412987	5.21 / 18326	5.32 / 19921	33	115	3	R.LVVLDEELEGISPDELKDELPER.Q	26

CAPNS1 Calpain small subunit 1	IPI00025084	5.05 / 28469	5.04 / 30107	33	126	5	R.FIVYSYK.Y<br>R.NTEDLTEEWLR.E<br>R.ILGGVISAISEAAAQYNPEPPPPR.T<br>R.THYSNIEANESEEVR.Q<br>K.TDGFGIDTCR.S<br>K.YLWNNIK.R<br>R.YSDESGNMDFDNFISCLVR.L Oxidation (M)	27
DECREASE								
STRAP Serine-threonine kinase receptor-associated protein	IPI00294536	4.98 / 38756	5.13 / 44724	33	56	2	K.SFEAPATINSASLHPEK.E<br>R.FSPDGELYASGSEDGTLR.L	10
Cell cycle/ Proliferation								
INCREASE								
MCM7 Isoform 1 of DNA replication licensing factor MCM7	IPI00299904	6.08 / 81884	6.48 / 92125	33	60	5	R.IAQPGDHVSVTGIFLPILR.T<br>K.ALLLLLVGGVDQSPR.G<br>R.SLEQNIQLPAALLSR.F<br>R.LAQHITYVHQHSR.Q<br>R.TQRPADVIFATVR.E	10
S100A11 Protein S100-A11	IPI00013895	6.56 / 11847	6.09 / 14087	33	51	2	K.TEFLSFMNTELAAFTK.N<br>K.TEFLSFMNTELAAFTK.N Oxidation (M)	15
Unknown								
DECREASE								
C19orf10 UPF0556 protein C19orf10 precursor	IPI00056357	6.2 / 18897	6.63 / 16785	32	52	2	K.SYLYFTQFK.A<br>R.ESDVPLKTEEFEVTK.T	13
MTPN Myotrophin	IPI00179589	5.92 / 14508	5.2 / 14754	32	226	3	K.GADINAPDKHHITPLLSAVYEGHVSCVK.L<br>K.HHITPLLSAVYEGHVSCVK.L<br>K.GPDGLTAFEATDNQAIK.A	34
C1QBP Complement component 1 Q subcomponent-binding protein, mitochondrial	IPI00014230	4.74 / 31742	4.42 / 31654	32	105	3	K.AFVDFLSDEIKEER.K<br>K.VEEQEPELTSTPNFVVEVIK.N	21

	K.ALVLDCHYPEDEVGQEDEAESDIFSIR.E

[a] Accession numbers of protein were derived from the International Protein Index (IPI) database.

[b] Protein's sequence coverage by peptide identification.
