# Supplementary figures and images for: A comparative proteomic study identified LRPPRC and MCM7 as putative actors in imatinib mesylate cross-resistance in Lucena cell line
Source: Proteome Sci. 2012 Mar 30;10:23. doi: 10.1186/1477-5956-10-23 (PMC3361502; doi:10.1186/1477-5956-10-23)

## ROC CURVE

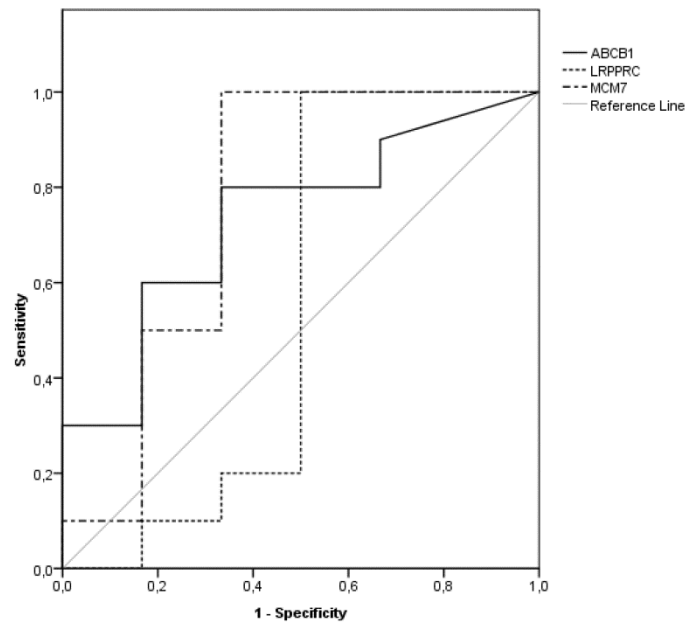

Supplement: Additional file 2 — ROC curve. ROC curve analysis showed that ABCB1. LRPPRC and MCM7 genes, together, are potential candidate biomarkers for IM therapy response for further investigation. Area under curve (AUC): 0.733 (ABCB1); 0.550 (LRPPRC); 0.767 (MCM7). [file 1477-5956-10-23-S2.PDF]
